# Efficacy of treatment patterns based on concurrent chemoradiotherapy in patients with stage IIB cervical squamous cell carcinoma

**DOI:** 10.1186/s12885-023-11372-6

**Published:** 2024-01-18

**Authors:** Xin-Bin Pan, Yan Lu, You-Sheng Wei, De-Sheng Yao

**Affiliations:** 1 Department of Radiation Oncology, Guangxi Medical University Cancer Hospital, 530021 Nanning, Guangxi P.R. China; 2 Department of Gynecologic Oncology, Guangxi Medical University Cancer Hospital, 530021 Nanning, Guangxi P.R. China; 3 No. 71 Hedi Road, Qingxiu District, 530021 Nanning, Guangxi P.R. China

**Keywords:** Cervical squamous cell carcinoma, CSCC, Radiotherapy, Chemotherapy, Stage IIB

## Abstract

**Purpose:**

To assess survival of treatment patterns based on concurrent chemoradiotherapy (CCRT) in patients with stage IIB cervical squamous cell carcinoma (CSCC).

**Materials and methods:**

Patients with stage IIB CSCC receiving CCRT were investigated from June 2012 to June 2019 in Guangxi Medical University Cancer Hospital. Baseline characteristics and treatment patterns were described. Survival between treatment patterns were compared using Kaplan-Meier methods.

**Results:**

A total of 232 patients were included: 39.7% of patients received CCRT alone, 6.5% of patients received neoadjuvant chemotherapy (NACT) + CCRT, 45.6% of patients received CCRT + adjuvant chemotherapy (AC), and 8.2% of patients received NACT + CCRT + AC. CCRT + AC showed similar overall survival (OS; hazard ratio [HR] = 0.95, 95% confidence interval [CI]: 0.41–2.17; *P* = 0.894) and locoregional-free survival (LRFS; HR = 2.39, 95% CI: 0.45–12.63; *P* = 0.303) compared with CCRT. However, CCRT + AC had a worse distant metastasis-free survival (DMFS; HR = 5.39, 95% CI: 1.14–25.57; *P* = 0.034). After propensity score matching, CCRT + AC had comparable OS (HR = 0.89, 95% CI: 0.29–2.70; *P* = 0.833), LRFS (HR = 3.26, 95% CI: 0.30-35.38; *P* = 0.331), and DMFS (HR = 4.80, 95% CI: 0.55–42.26; *P* = 0.157) compared to CCRT.

**Conclusion:**

AC did not improve survival in patients with stage IIB CSCC receiving CCRT.

**Supplementary Information:**

The online version contains supplementary material available at 10.1186/s12885-023-11372-6.

## Introduction

Cervical squamous cell carcinoma (CSCC) is a major health threat of women worldwide [1]. In developing countries, patients usually present with locally advanced diseases [2]. Concurrent chemoradiotherapy (CCRT) is the standard treatment for these patients [3]. However, approximately 17% of patients experienced local recurrences and 18% of patients developed distant metastases [4–6].

Neoadjuvant chemotherapy (NACT) and adjuvant chemotherapy (AC) were expected to improve local control and reduce distant metastasis. However, studies investigating NACT and AC combined with CCRT have yielded inconsistent results [7–19]. The optimal treatment strategy remains uncertain, especially in the stage IIB subgroup. This study aims to evaluate treatment patterns and outcomes in patients with stage IIB CSCC.

## Materials and methods

### Patients

We identified CSCC patients who were treated at Guangxi Medical University Cancer Hospital from June 2012 to June 2019. Inclusion criteria were as follows: [1] pathologically confirmed cervical cancer, [2] stage IIB according to the FIGO staging system, and [3] squamous cell carcinoma. Exclusion criteria were as follows: [1] patients refused treatments, [2] patients had incomplete data, [3] patients did not finish treatments, and [4] patients received surgery.

Clinical characteristics (age, Eastern Cooperative Oncology Group [ECOG] performance status, tumor grade, hemoglobin, human papilloma virus [HPV] infection status, tumor diameter, and concurrent chemotherapy [CCT] cycles) and treatment patterns were extracted.

### Treatments

Patients underwent pelvic external beam radiotherapy in combination with high-dose-rate intracavitary brachytherapy. The pelvic external beam radiotherapy involved a dose of 48–50 Gy delivered in 24–25 fractions using intensity-modulated radiotherapy. The high-dose-rate intracavitary brachytherapy was given at 28–35 Gy delivered in 4–5 fractions to the high-risk clinical target volume.

Platinum-based NACT was administered every 3 weeks before CCRT. The CCT consisted of either cisplatin at 30–40 mg/m^2^ on day 1 or nedaplatin at 50 mg/m^2^ on day 1 per week, during the course of radiotherapy. After CCRT, platinum-based AC was administered every 3 weeks.

### Endpoints

Treatment failures were identified based on records, including pathology reports and/or imaging reports. Death events were determined from official statements.

The primary endpoint of the study was overall survival (OS). OS was defined as the duration from the date of diagnosis to the date of death due to any cause. The secondary endpoints were locoregional-free survival (LRFS), which was defined as the duration from the date of diagnosis to the date of locoregional recurrence, and distant metastasis-free survival (DMFS), which was defined as the duration from the date of diagnosis to the date of distant metastasis.

### Statistical analysis

The continuous variable of tumor diameter was categorized based on a threshold of 4 cm [20]. Similarly, the continuous variables of age and hemoglobin were transformed into categorical variables using their respective median values. Categorical variables, including age, ECOG, tumor grade, hemoglobin, HPV infection status, tumor diameter, and CCT cycles were analyzed using the χ^2^ test or Fisher’s exact test.

For the analysis of OS, LRFS, and DMFS between treatment patterns, the Kaplan-Meier method with log-rank test statistics was employed. Pairwise comparisons were conducted among the different treatment patterns. The identification of independent prognostic factors was carried out using multivariable proportional hazards regressions, which adjusted for factors including age, ECOG, tumor grade, hemoglobin, HPV infection status, tumor diameter, and treatment patterns. The results were recorded as hazard ratios (HRs) with corresponding 95% confidence intervals (CIs).

To mitigate selection bias between CCRT and CCRT + AC subgroups, a matched case-control analysis was performed using propensity score matching (PSM). Patients who received CCRT were considered the dependent variable in calculating the propensity scores. One-to-one matching without replacement was implemented in the logistic regression model, utilizing a caliper of 0.02 on the logit of the propensity score.

This study used SPSS Statistics Version 26.0 software (IBM Co., Armonk, NY, USA) and R software (version 4.2.1) to perform statistical analyses. Two-tailed *P* values <  0.05 were considered statistically significant.

Ethical approval for this study was obtained from the Guangxi Medical University Cancer Hospital Ethics Committee. The study was conducted in compliance with the principles outlined in the Declaration of Helsinki. However, informed consent was not obtained due to the retrospective nature of the study.

## Results

### Baseline characteristics

**Fig. 1:**
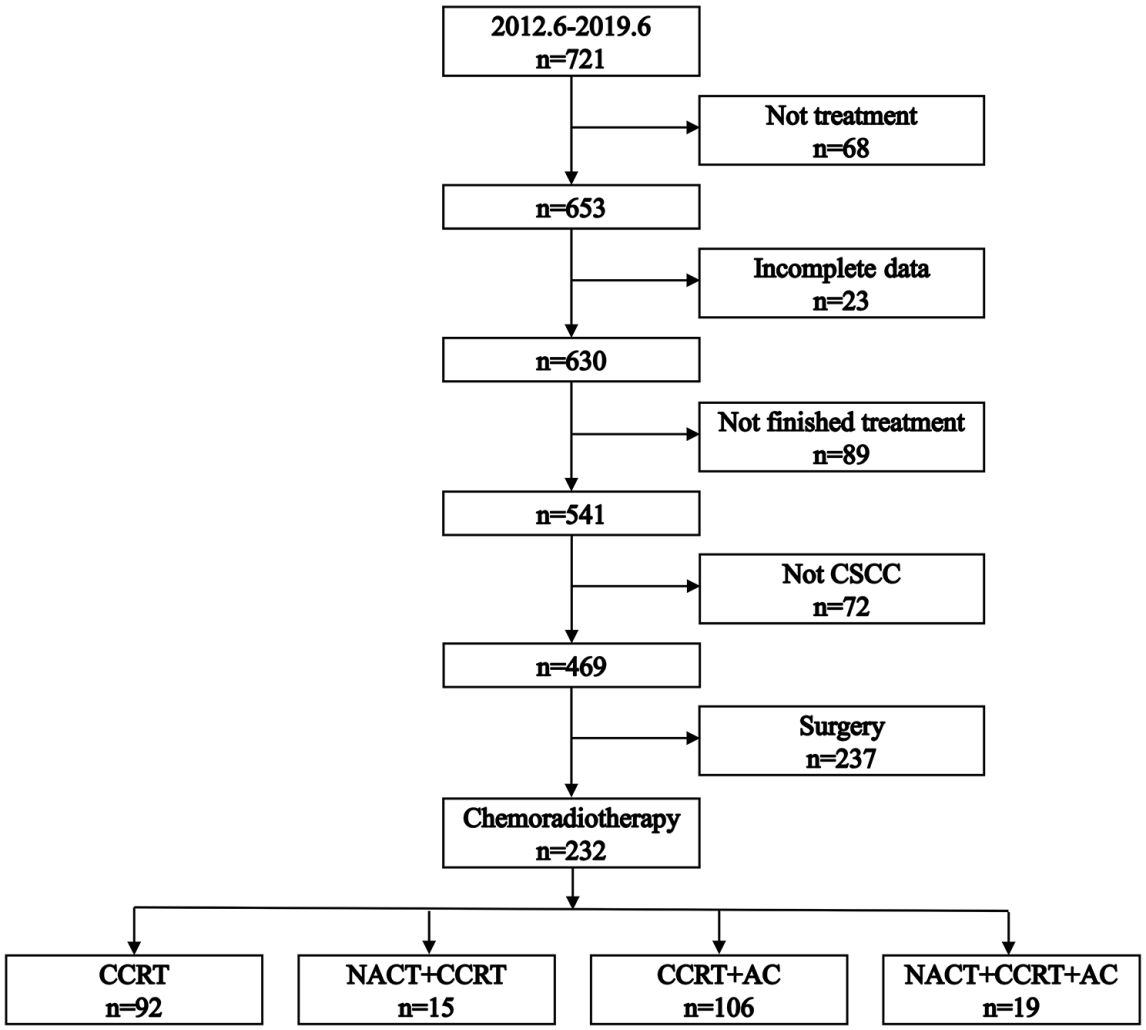
Flowchart depicting patient selection. CSCC: cervical squamous cell carcinoma. CCRT: concurrent chemoradiotherapy. AC: adjuvant chemotherapy. NACT: neoadjuvant chemotherapy

Figure 1 illustrates the patient selection process, wherein 232 patients were included after investigating a total of 721 patients. Table 1 provides a summary of the patient characteristics. The last follow-up time was October 2021. The median follow-up time was 54 months (interquartile range: 37–77 months). Thirty patients were lost to follow-up, resulting in a follow-up rate of 87.1%.

**Table 1:** Patient characteristics

	CCRT (n = 92)	NACT + CCRT (n = 15)	CCRT + AC (n = 106)	NACT + CCRT + AC (n = 19)	*P*
Age (year)					< 0.001
≤54	32 (34.8%)	8 (53.3%)	73 (68.9%)	16 (84.2%)	
>54	60 (65.2%)	7 (46.7%)	33 (31.1%)	3 (15.8%)	
ECOG					0.083
0	19 (20.7%)	2 (13.3%)	36 (34.0%)	7 (36.8%)	
1	73 (79.3%)	13 (86.7%)	70 (66.0%)	12 (63.2%)	
Grade					0.587
I	1 (1.0%)	0 (0.0%)	2 (1.8%)	0 (0.0%)	
II	24 (26.1%)	7 (46.7%)	25 (23.6%)	4 (21.1%)	
III	41 (44.6%)	7 (46.7%)	45 (42.5%)	8 (42.1%)	
unknown	26 (28.3%)	1 (6.6%)	34 (32.1%)	7 (36.8%)	
Hgb (g/L)					0.258
≤117	44 (47.8%)	10 (66.7%)	54 (50.9%)	13 (68.4%)	
>117	48 (52.2%)	5 (33.3%)	52 (49.1%)	6 (31.6%)	
HPV					0.212
negative	9 (9.7%)	0 (0.00%)	13 (12.3%)	1 (5.3%)	
positive	65 (70.7%)	9 (60.0%)	79 (74.5%)	13 (68.4%)	
unknown	18 (19.6%)	6 (40.0%)	14 (13.2%)	5 (26.3%)	
Tumor-diameter					0.034
≤4	38 (41.3%)	7 (46.7%)	25 (23.6%)	6 (31.6%)	
>4	54 (58.7%)	8 (53.3%)	81 (76.4%)	13 (68.4%)	

ECOG: Eastern Cooperative Oncology Group. Hgb: hemoglobin. HPV: human papilloma virus. CCRT: concurrent chemoradiotherapy. AC: adjuvant chemotherapy. NACT: neoadjuvant chemotherapy

### Treatment patterns

The treatment patterns investigated in this study included CCRT, NACT + CCRT, CCRT + AC, and NACT + CCRT + AC. The most commonly used treatment modalities were CCRT, accounting for 39.7% of the patients, and CCRT + AC, accounting for 45.7% of the patients. However, the sample sizes for the NACT + CCRT (6.5%) and NACT + CCRT + AC (8.1%) subgroups were relatively limited.

The CCRT subgroup had a higher proportion of patients with age > 54 years, while the AC subgroup had a higher proportion of patients with tumor diameter > 4 cm. However, factors including ECOG, tumor grade, hemoglobin level, and HPV infection status did not exhibit significant differences among the various treatment patterns.

### Overall survival

**Fig. 2:**
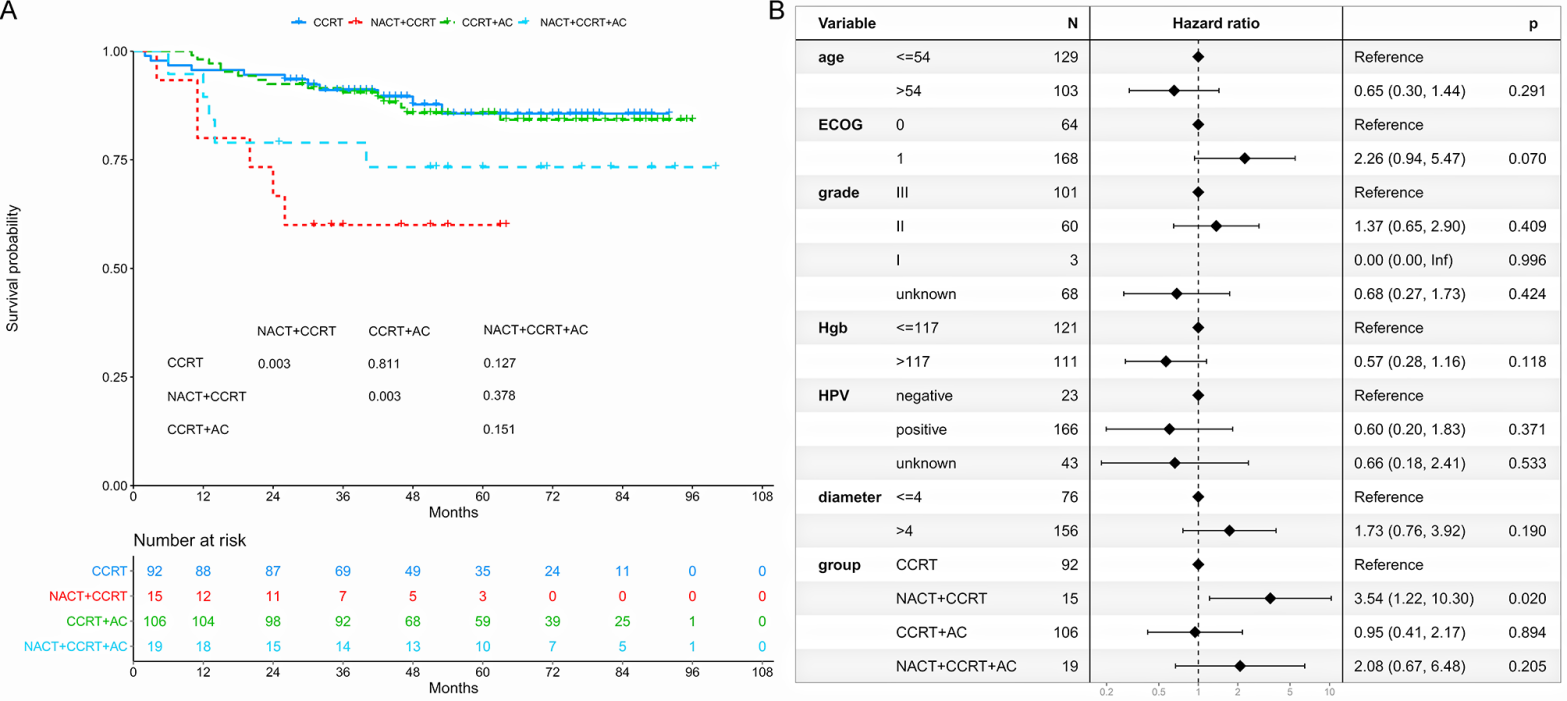
Overall survival of treatment patterns. (**A**) Overall survival between the CCRT, NACT + CCRT, CCRT + AC, and NACT + CCRT + AC subgroups. (**B**) Multivariable proportional hazards regressions of overall survival. CCRT: concurrent chemoradiotherapy. AC: adjuvant chemotherapy. NACT: neoadjuvant chemotherapy

The 5-year OS was 85.6%, 60.0%, 85.8%, and 73.3% for CCRT, NACT + CCRT, CCRT + AC, and NACT + CCRT + AC groups, respectively (Fig. 2A). NACT + CCRT subgroup had a worse 5-year OS compared to both the CCRT (*P* = 0.003) and CCRT + AC (*P* = 0.003) subgroups. However, there was no difference in the 5-year OS between the CCRT and CCRT + AC subgroups (*P* = 0.811). Multivariate regression analysis revealed that NACT + CCRT was identified as an independent prognostic factor for OS (HR = 3.54, 95% CI: 1.22–10.30; *P* = 0.020; Fig. 2B).

### Locoregional-free survival

**Fig. 3:**
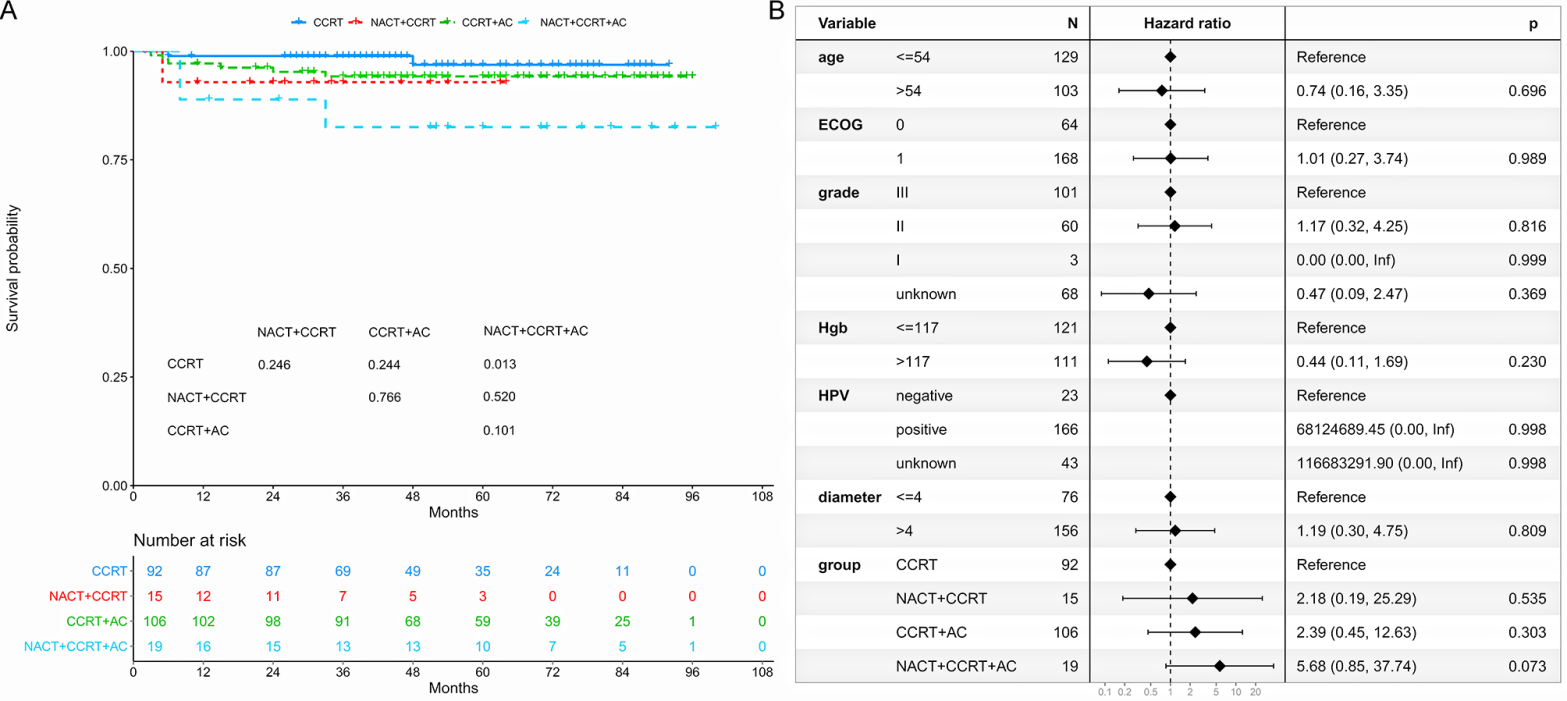
Locoregional-free survival of treatment patterns. (**A**) Locoregional-free survival between the CCRT, NACT + CCRT, CCRT + AC, and NACT + CCRT + AC subgroups. (**B**) Multivariable proportional hazards regressions of locoregional-free survival. CCRT: concurrent chemoradiotherapy. AC: adjuvant chemotherapy. NACT: neoadjuvant chemotherapy

The 5-year LRFS was 96.9%, 92.9%, 94.2%, and 82.5% for CCRT, NACT + CCRT, CCRT + AC, and NACT + CCRT + AC subgroups, respectively (Fig. 3A). The NACT + CCRT + AC subgroup exhibited a worse 5-year LRFS compared to the CCRT subgroup (*P* = 0.013). However, there were no differences in the 5-year LRFS between the CCRT, NACT + CCRT, and CCRT + AC subgroups. Multivariate regression analysis revealed that NACT + CCRT + AC was not identified as an independent prognostic factor for LRFS (HR = 5.68, 95% CI: 0.85–37.74; *P* = 0.073; Fig. 3B).

### Distant metastasis-free survival

**Fig. 4:**
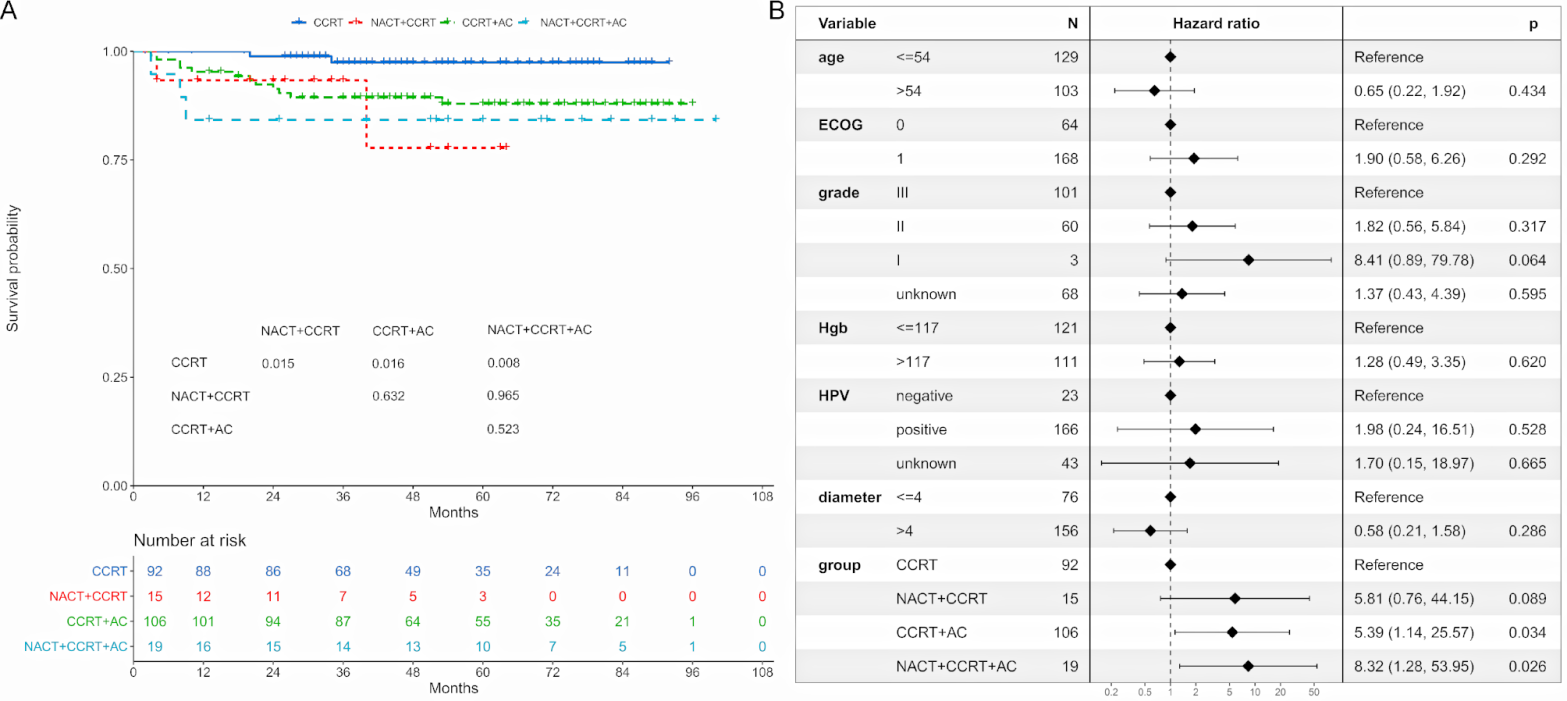
Distant metastasis-free survival of treatment patterns. (**A**) Distant metastasis-free survival between the CCRT, NACT + CCRT, CCRT + AC, and NACT + CCRT + AC subgroups. (**B**) Multivariable proportional hazards regressions of distant metastasis-free survival. CCRT: concurrent chemoradiotherapy. AC: adjuvant chemotherapy. NACT: neoadjuvant chemotherapy

The 5-year DMFS was 97.4%, 77.8%, 87.9%, and 84.2% for CCRT, NACT + CCRT, CCRT + AC, and NACT + CCRT + AC subgroups, respectively (Fig. 4A). The CCRT subgroup had better 5-year DMFS rates compared to the NACT + CCRT (*P* = 0.015), CCRT + AC (*P* = 0.016), and NACT + CCRT + AC (*P* = 0.008) subgroups. Multivariate regression analysis revealed that both CCRT + AC (HR = 5.39, 95% CI: 1.14–25.57; *P* = 0.034) and NACT + CCRT + AC (HR = 8.32, 95% CI: 1.28–53.95; *P* = 0.026) were identified as independent prognostic factors for DMFS (Fig. 4B).

### Survivals between CCRT and CCRT + AC subgroups after PSM

**Fig. 5:**
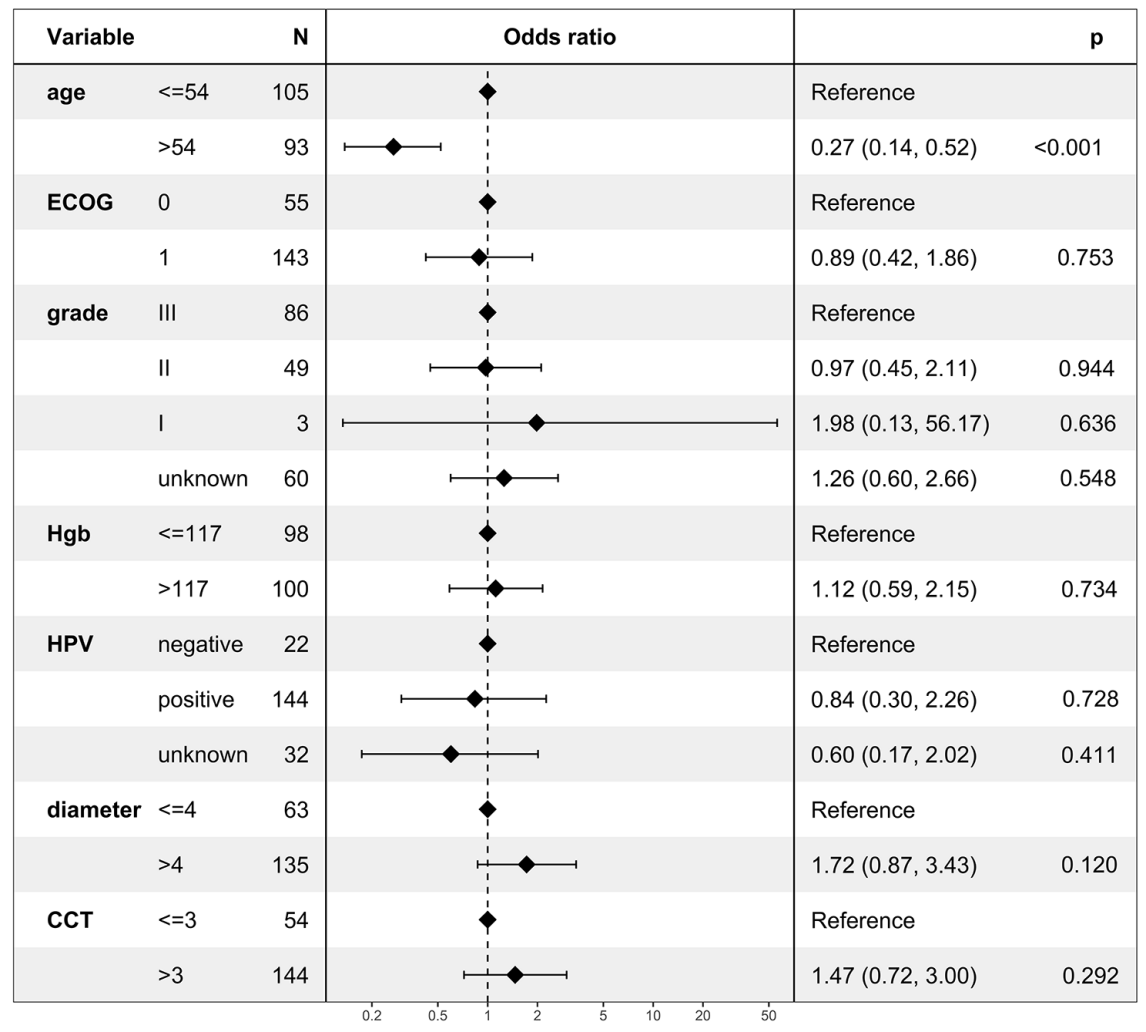
Logistic regression analysis for factors associated with adjuvant chemotherapy use. ECOG: Eastern Cooperative Oncology Group. Hgb: hemoglobin. HPV: human papilloma virus. CCT: concurrent chemotherapy

In the multivariate logistic regression analysis, it was observed that patients with age > 54 years were less likely to receive CCRT + AC (odds ratio = 0.27, 95% CI: 0.14–0.52; P < 0.001; Fig. 5). After PSM, 55 patients who received CCRT and 55 patients who received CCRT + AC were matched. Table 2 summarizes the patient characteristics after PSM. The patient characteristics were found to be well-balanced across all covariates after PSM (*P* > 0.05).

**Table 2:** Patient characteristics of CCRT and CCRT + AC subgroups after propensity score matching

	CCRT (n = 55)	CCRT + AC (n = 55)	*P*
Age (year)			0.849
≤54	28 (50.9%)	30 (54.5%)	
>54	27 (49.1%)	25 (45.5%)	
ECOG			0.284
0	18 (32.7%)	12 (21.8%)	
1	37 (67.3%)	43 (78.2%)	
Grade			0.398
II	13 (23.6%)	11 (20.0%)	
III	21 (38.2%)	28 (50.9%)	
unknown	21 (38.2%)	16 (29.1%)	
Hgb (g/L)			0.567
≤117	27 (49.1%)	31 (56.4%)	
>117	28 (50.9%)	24 (43.6%)	
HPV			0.381
negative	7 (12.7%)	3 (5.5%)	
positive	42 (76.4%)	44 (80.0%)	
unknown	6 (10.9%)	8 (14.5%)	
Tumor-diameter			0.690
≤4	21 (38.2%)	18 (32.7%)	
>4	34 (61.8%)	37 (67.3%)	
CCT cycles			0.829
≤3	15 (27.3%)	14 (25.5%)	
>3	40 (72.7%)	41 (74.5%)	

ECOG: Eastern Cooperative Oncology Group. Hgb: hemoglobin. HPV: human papilloma virus. CCRT: concurrent chemoradiotherapy. CCT: concurrent chemotherapy. AC: adjuvant chemotherapy

**Fig. 6:**
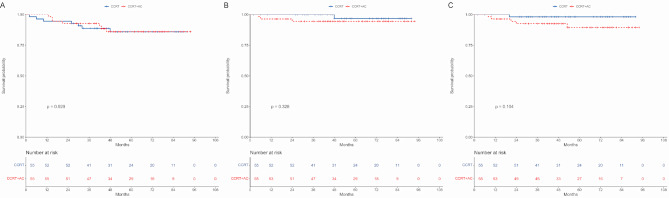
Survivals between CCRT and CCRT + AC subgroups after propensity score matching. (**A**) Overall survival. (**B**) Locoregional-free survival. (**C**) Distant metastasis-free survival. CCRT: concurrent chemoradiotherapy. AC: adjuvant chemotherapy

CCRT + AC did not improve the 5-year OS (85.9% vs. 86.0%; *P* = 0.920, Fig. 6A), LRFS (96.8% vs. 94.5%; *P* = 0.328, Fig. 6B), or DMFS (98.1% vs. 89.4%; *P* = 0.104, Fig. 6C) compared to CCRT. Multivariate regression analysis revealed that CCRT + AC was not identified as an independent prognostic factor for OS (HR = 0.89, 95% CI: 0.29–2.70; *P* = 0.833), LRFS (HR = 3.26, 95% CI: 0.30-35.38; *P* = 0.331), or DMFS (HR = 4.80, 95% CI: 0.55–42.26; *P* = 0.157) (Table 3).

**Table 3:** Multivariable proportional hazards regressions of survivals between CCRT and CCRT + AC subgroups after propensity score matching

	OS			LRFS			DMFS		
	HR	95% CI	*P*	HR	95% CI	*P*	HR	95% CI	*P*
Age (year)									
≤54	reference			reference			reference		
>54	0.75	0.19–2.87	0.672	1.35	0.36-502.33	0.159	1.86	0.26–13.38	0.540
ECOG									
0	reference			reference			reference		
1	1.79	0.45–7.13	0.409	1.08	0.01–2.04	0.138	1.85	0.15–23.21	0.632
Grade									
III	reference			reference			reference		
II	1.61	0.35–7.53	0.542				1.96	0.26–15.10	0.517
unknown	1.50	0.37–6.04	0.571	0.33	0.02–6.68	0.469	1.21	0.15–9.83	0.861
Hgb (g/L)									
≤117	reference			reference			reference		
>117	0.73	0.19–2.74	0.637	0.15	0.01–2.74	0.200	2.10	0.29–15.12	0.462
HPV									
negative	reference			reference			reference		
positive	0.22	0.05–1.09	0.063						
unknown	0.51	0.06–4.06	0.521						
Tumor-diameter									
≤4	reference			reference			reference		
>4	2.40	0.63–9.08	0.197	0.99	0.06–16.65	0.993	0.28	0.05–1.69	0.165
CCT cycles									
≤3	reference			reference			reference		
>3	0.67	0.18–2.62	0.573				1.12	0.16–8.57	0.915
Group									
CCRT	reference			reference			reference		
CCRT + AC	0.89	0.29–2.70	0.833	3.26	0.30-35.38	0.331	4.80	0.55–42.26	0.157

ECOG: Eastern Cooperative Oncology Group. Hgb: hemoglobin. HPV: human papilloma virus. CCT: concurrent chemotherapy. CCRT: concurrent chemoradiotherapy. AC: adjuvant chemotherapy. HR: hazard ratio. CI: confidence interval. OS: overall survival. LRFS: locoregional-free survival. DMFS: distant metastasis-free survival

## Discussion

This study revealed two main findings. First, the most common treatment modalities for stage IIB CSCC were CCRT and CCRT + AC. Second, the addition of chemotherapy before or after CCRT did not improve survival for patients with stage IIB CSCC. Consequently, well-designed prospective, randomized controlled trials are needed to explore alternative treatments that may enhance survival rates in this patient population.

NACT can inhibit cancer cells implantation and eliminate cancer cells in the circulation, thus reducing subclinical metastasis. Additionally, NACT can effectively decrease the tumor load in the local and regional areas, ultimately leading to an increase in the rate of locoregional tumor control. However, despite its potential benefits, previous studies have reported that NACT may result in decreased disease-free survival and OS in patients with locally advanced diseases, [11, 18] particularly in stage IIB diseases [21, 22]. Our study also yielded similar findings, where patients receiving NACT exhibited worse OS (*P* = 0.003), LRFS (*P* = 0.013), and DMFS (*P* = 0.015) in comparison to those who underwent CCRT.

The reasons behind the detrimental effect of NACT in some cases remain unclear. Several possible explanations have been proposed: First, the delay caused by administering NACT before CCRT could potentially decrease survival rates. The time lapse between the two treatments may allow the cancer to progress or become more aggressive, affecting patient outcomes [23]. Second, cancer cells may acquire resistance to the treatment during the course of NACT. This resistance could make the cancer more difficult to control or eliminate during subsequent CCRT [24]. Third, NACT may lead to significant toxicity in some patients, which could affect their ability to tolerate and complete subsequent CCRT. The adverse events associated with NACT might interfere with the optimal delivery of subsequent CCRT, impacting treatment efficacy [18].

Due to the current limited data and conflicting findings from previous studies, further investigation is warranted. The ongoing head-to-head phase III INTERLACE trial (ClinicalTrials.gov identifier: NCT01566240) is specifically designed to evaluate the efficacy of NACT in patients with locally advanced diseases. It will provide a more comprehensive understanding of the role of NACT in the management of this patient population.

AC aims to eliminate potential residual tumor, both within the pelvis and beyond. A meta-analysis reported that CCRT + AC was associated with improved OS (HR = 0.78, 95% CI: 0.69–0.88; *P* < 0.0001) and progression-free survival (HR = 0.80, 95% CI: 0.73–0.87; *P* < 0.0001) compared to CCRT [7]. However, outcomes of AC were not consistent across studies. Two phase III trials (ACTLACC and OUTBACK trials) demonstrated that adjuvant carboplatin and paclitaxel chemotherapy did not improve OS but led to increased toxicity when compared to CCRT [14, 15].

Several possible explanations for the inconsistent results of AC are as follows: First, studies included different pathological subtypes [7, 8]. The efficacy of AC was different among different histological subtypes (squamous cell carcinoma and adenocarcinoma) [25]. Second, the paclitaxel plus carboplatin chemotherapy regimen may not be effective [26–28]. Third, studies included different FIGO stages [7, 8]. The benefits of AC might differ among different FIGO stages [26]. Due to these variations and potential confounding factors, efficacy of AC in patients with locally advanced diseases needs further assessment.

Our study revealed that AC did not improve survivals in patients with stage IIB CSCC. The result was consistent with the results from the OUTBACK trial [14]. One possible explanation for this lack of benefit was that stage IIB disease has a relatively lower tumor burden compared to other locally advanced diseases. As a result, CCRT may already provide satisfactory treatment outcomes in this subgroup of patients. In contrast, AC leads to an increase in treatment-related toxicities. These adverse events could potentially impact patient survival adversely [15]. Furthermore, patients should be divided into different risk subgroups based on various prognostic factors. AC may be more beneficial for high-risk patients, while it may not provide significant advantages for low-risk patients [29].

A major limitation of this study should be considered. The sample sizes of NACT + CCRT and NACT + CCRT + AC subgroups were quite small. Small sample sizes can limit the statistical power to detect significant differences in survival outcomes between treatment patterns. Although efforts were made to adjust for all the factors, including age, ECOG, tumor grade, hemoglobin, HPV infection status, tumor diameter, and treatment patterns through multivariable proportional hazards regressions, potential unmeasured statistical biases might still exist. These biases could influence the conclusions and interpretations of this study. To address this limitation and further validate the findings, large sample size randomized controlled trials are needed.

In conclusion, our study suggested that AC did not improve treatment outcomes in patients with stage IIB CSCC receiving CCRT.

### Electronic supplementary material

Below is the link to the electronic supplementary material.

Supplementary Material 1

## Data Availability

The datasets used and/or analyzed during the current study are available from the Supplemental file.

## List of abbreviations

CCRT: concurrent chemoradiotherapy
CSCC: cervical squamous cell carcinoma
NACT: neoadjuvant chemotherapy
AC: adjuvant chemotherapy
OS: overall survival
LRFS: locoregional-free survival
DMFS: distant metastasis-free survival
HR: hazard ratio
CI: confidence interval
